# ISG15 Inhibits IFN-****α****-Resistant Liver Cancer Cell Growth

**DOI:** 10.1155/2013/570909

**Published:** 2013-08-20

**Authors:** Xin-xing Wan, Han-chun Chen, Md. Asaduzzaman Khan, Ai-hua Xu, Fu-lan Yang, Yun-yi Zhang, Dian-zheng Zhang

**Affiliations:** ^1^Department of Biochemistry, School of Life Sciences, Central South University, Changsha, Hunan 410013, China; ^2^Department of Biochemistry/Molecular Biology, Philadelphia College of Osteopathic Medicine, Philadelphia, PA 19131, USA

## Abstract

Hepatocellular carcinoma (HCC) is one of the most prevalent tumors worldwide. Interferon-**α** (IFN-**α**) has been widely used in the treatment of HCC, but patients eventually develop resistance. ISG15 ubiquitin-like modifier (ISG15) is a ubiquitin-like protein transcriptionally regulated by IFN-**α** which shows antivirus and antitumor activities. However, the exact role of ISG15 is unknown. In the present study, we showed that IFN-**α** significantly induced ISG15 expression but failed to induce HepG2 cell apoptosis, whereas transient overexpression of ISG15 dramatically increased HepG2 cell apoptosis. ISG15 overexpression increased overall protein ubiquitination, which was not observed in cells with IFN-**α**-induced ISG15 expression, suggesting that IFN-**α** treatment not only induced the expression of ISG15 but also inhibited ISG15-mediated ubiquitination. The tumor suppressor p53 and p21 proteins are the key regulators of cell survival and death in response to stress signals such as DNA damage. We showed that p53 or p21 is only up regulated in HepG2 cells ectopically expressing ISG15, but not in the presence of IFN-**α**-induced ISG15. Our results suggest that ISG15 overexpression could be developed into a powerful gene-therapeutic tool for treating IFN-**α**-resistant HCC.

## 1. Introduction

Hepatocellular carcinoma (HCC) is one of the most prevalent tumors worldwide and a major threat to our modern life [1]. Although different therapies are available, interferon-*α* (IFN-*α*) is considered one of the treatments of choice for patients with hepatic cancer. IFN-*α* not only retards cancer cell growth by inhibiting angiogenesis and proliferation, but also it modulates the immune response and promotes apoptosis. However, patients often develop resistance to IFN-*α*, and the mechanisms of resistance are unknown. Therefore, the development of efficient strategies for overcoming resistance to IFN-*α* in HCC patients is essential [2].

IFN-*α* signaling pathway, which has been studied extensively, is activated by the phosphorylation of Janus kinase 1 (JAK1) and tyrosine kinase 2 (TYK2), which activates the downstream signal transducer and activator of transcription 1 (Stat1) and Stat2, leading to the formation of the transcriptional complex ISG factor 3 (ISGF3) [3]. ISGF3 upregulates the expression of a wide spectrum of genes which contain the IFN-*α*-stimulated response elements (ISREs) in their promoters [4, 5]. ISG15 is a ubiquitin-like protein whose expression is induced by IFN-*α* treatment [6]. Protein modification by ISG15 is known as ISGylation and occurs through sequential reactions similar to ubiquitination. Ubiquitination is a type of protein modification that consists of the attachment of ubiquitin to specific lysine residues of the target substrate. Proteins can be mono-, multi-, or polyubiquitinated, and poly-ubiquitination usually leads to protein degradation via the 26S proteasome system [7, 8]. ISGylation, which affects the stability, subcellular localization and function of the modified proteins, is mediated by an E1 activating enzyme (Ube1l), E2 conjugating enzymes (UbcH8, UbcH6), and E3 ligases (EFP) [9–11]. Hundreds of proteins have been identified as targets of ISGylation, and protein modification by ISGylation has been widely shown to be involved in the regulation of different biological processes included in cancer development and therapy [12].

Although both ISG15 and the ISGlyation enzymes are regulated by IFN-*α*, the effects of ISG15 and ISGlyation on IFN-*α* therapy and their roles in IFN-*α* resistance are unknown. We used the HepG2 cell line as a model system to study the effects of ISG15 expression on cancer cell apoptosis. As HepG2 cells express the wild-type form of the tumor suppressor p53 [13], this model system also allowed us to detect the effects of ISG15 on p53 and its downstream protein p21, both of which play the key regulatory roles in cell survival or cell death [14, 15].

## 2. Materials and Methods

### 2.1. Cell Culture and IFN-*α* Treatment

HepG2 cells were cultured in RPMI1640 medium (Invitrogen, USA) supplemented with 10% fetal bovine serum (Sijiqing Biological Engineering Materials Co., Ltd., China) at 37°C in an atmosphere with 5% CO_2_. IFN-*α* treatment was performed in 5 mL growth medium with different concentrations (0, 500, 1000, and 2000 U/mL) of IFN-*α* for 48 h.

### 2.2. Plasmid Construction and Transfection

Human ISG15 (Gene ID: 9636) cDNA was made by the reverse transcriptase reaction from total RNA extracted from IFN-*α*-treated human chronic myeloid leukemia cell line KT1-A3 cells. The PCDNA3.1-ISG15 plasmid was constructed by inserting the ISG15 cDNA amplified by RT-PCR (upstream primer: CTTGGTACCGCAGCGAACTCATCTTTG and downstream primer: GGAGGATCCTCTTTACAACAGCCTTTATTTC) into the PCDNA3.1 plasmid (Invitrogen, NY, USA). The constructed plasmid was sequenced by BGI (a genomic research company, Beijing, China; web link: http://www.genomics.cn/en/index). HepG2 cells were seeded in 6-well plates (3 × 10^5^ cells/well) and transfected using Sofast Transfection Reagent (Sunma Biotech Corp., Xiamen, China). The medium was replaced by fresh RPMI1640 after 6 h and incubated for 12 h followed by further experiments.

### 2.3. Western Blotting Analysis

The harvested cells were lysed by cold RIPA buffer (200 *μ*L/well, Beyotime Institute of Biotechnology Corp., Shanghai, China) supplemented with 1 mM proteinase inhibitor PMSF (Beyotime Insititute of Biotechnology Corp., Shanghai, China) for 30 min on ice, and then centrifuged at 15000 g for 15 min. The supernatant containing proteins were stored at −20°C. The protein concentration was determined by the Bradford assay. Proteins were separated by sodium dodecyl sulphate-polyacrylamide gel electrophoresis (SDS-PAGE). After electrophoresis, proteins were transferred to nitrocellulose membrane by electroblotting and blocked in TBST (50 mM Tris-HCl, pH 7.5, 150 mM NaCl, 0.2% Tween-20) containing 5% nonfat milk for 2 h at room temperature. The blots were incubated with the primary antibody (1 : 1000) at 4°C overnight and washed with TBST three times. Then, the blots were incubated with the secondary antibody (1 : 5000) at room temperature for 1 h. All the antibodies used in this study (ISG15, p53, ubiquitin, *β*-actin, and horseradish peroxidase-conjugated secondary antibodies) were purchased from Cell Signaling Technology, USA. 

### 2.4. Subcellular Localization by Confocal-Scanning Laser Microscopy

The cells with different treatments were fixed in 4% paraformaldehyde and permeabilized with 0.1% Triton-X100 in PBS containing 1% BSA for 10 min. The washed cells were then blocked in PBS containing 10% BSA at 37°C for 30 min and incubated with ISG15 primary antibody (1 : 50) at 4°C overnight. The cells were then washed with PBS and incubated with Cy5-labeled secondary antibody (1 : 100) at room temperature for 1 h. After washing again, the cells were dyed by DAPI and watched by using confocal-scanning laser microscopy.

### 2.5. Flow Cytometric Analysis

Cells were collected, washed twice with PBS (pH 7.4), and fixed in 70% ethanol overnight. Then, the fixed cells were washed with 1 mL of PBS. Then 500 *μ*L of PBS containing 3, 8-diamino-5-[3-(diethylmethylammonio)propyl]-6-phenylphenanthridinium diiodide (PI, 50 *μ*g/mL) and RNase A (100 *μ*g/mL) was added at 37°C in dark room. After 30 min, the cells were analyzed by Cytomics FC500 (Beckman Coulter, USA). The data were collected by the software of CXP, and results were analyzed by ModFit LT. Flow cytometric analysis was performed by DingGuo Company (Beijing DingGuo ChangSheng Biotechnology Co., Ltd., China).

### 2.6. Statistical Analysis

Densitometry of western-blot bands were quantified by ImageJ software. Data were analyzed by one-way ANOVA and post hoc (least significant difference) LSD test. The statistical program SPSS 16.0 was used for the analysis, and the graphs were generated in MS Excel. Results were presented as mean ± SD (standard deviation). The star marker “*” indicates significant difference compared with control at *P* ≤ 0.05 level.

## 3. Results

### 3.1. IFN-*α* Induces ISG15 Expression in HepG2 Cells

**Figure 1:**
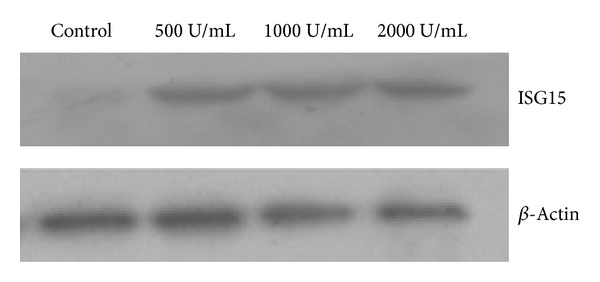
Expression of ISG15 in HepG2 cells. HepG2 cells were treated with different concentrations of IFN-*α* for 48 h. ISG15 expression was detected by western blotting using anti-ISG15 antibody.

HepG2 cells were treated with different concentrations of IFN-*α* for 48 h, and ISG15 levels were detected by western blotting (The experiment was repeated 3 times). As shown in Figure 1, ISG15 was undetectable in untreated HepG2 cells (control), and its levels were increased in response to IFN-*α* treatment, although a concentration-dependent effect (500 U/mL; 1000 U/mL; 2000 U/mL) was not observed. A dosage of 1000 U/mL of IFN-*α* was used in the subsequent experiments based on the commonly used dosage in clinical practice.

### 3.2. Effects of ISG15 on p53 and p21 Expression and Protein Ubiquitination

**Figure 2:**
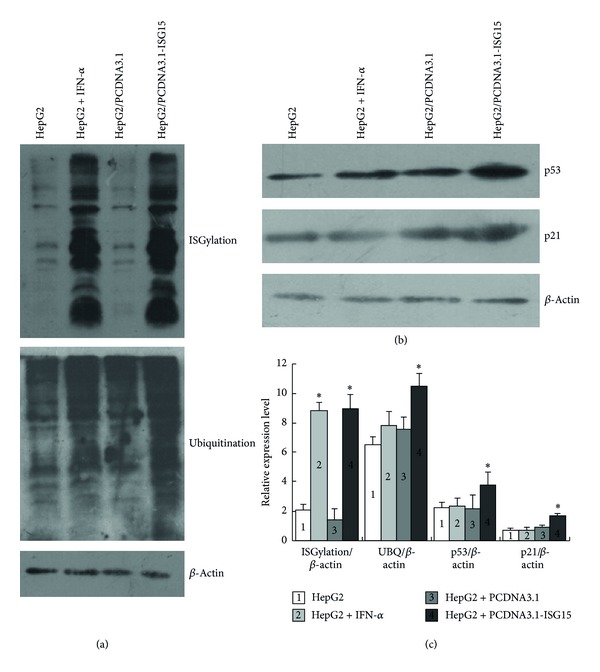
ISG15 overexpression induced ISGylation and ubiquitination and increased p53 levels (a) ISGylation and ubiquitination were detected by western blotting. HepG2 cells treated with IFN-*α* or transiently transfected with different plasmid were cultured for 48 h, and protein expression was assessed by western blotting. (b) p53 expression was examined by western blotting using anti-p53 antibody in cells treated with different factors for 48 h. (c) Densitometry of Western blotting bands was quantified by using ImageJ software (gray-scale band analysis). Data were analyzed by one-way ANOVA and post hoc LSD test. Results are shown as mean ± SD (*n* = 4). *P ≤ 0.05 compared with control HepG2 cells.

The increase of the levels of ISG15 in response to both IFN-*α* induction and transient transfection of cells with PCDNA3.1-ISG15 plasmid led us to examine whether ISGlyation was affected under these conditions. We quantified ISGylation, ubiquitination, and p53 and p21 levels for 4 times. Each time we recovered a new tube of HepG2 cells stored in our lab and cultured for experiments. Then, we added the IFN-*α* or transiently transfected PCDNA3.1-ISG15 and control PCDNA3.1 to divided cell independently. The statistical data of the densitometry of relative protein ratio of ISGylation/*β*-acitin, ubq/*β*-acitin, p53/*β*-acitin, and p21/*β*-acitin were used to identify whether the target proteins expression was changed (Figure 2(c)).

Overall protein ISGlyation was assessed by western blotting using specific antibody against ISG15. As shown in Figure 2(a), a basal level of protein ISGlyation was detected in the HepG2 cell lysate (Lane 1), which increased dramatically when the cells were treated with IFN-*α* (Lane 2) or transiently transfected with PCDNA3.1-ISG15 (Lane 4). Transient transfection of cells with the empty plasmid vector PCDNA3.1 had no effect on protein ISGlyation (Lane 3). Because of the similarity between ISGlyation and ubiquitination, we also analyzed the overall ubiquitination status in HepG2 cells treated with IFN-*α* and in those transfected with the PCDNA3.1-ISG15 plasmid. As shown in Figure 2(a), compared with the basal levels of protein ubiquitination (Lane 1), IFN-*α*-induced ISG15 also increased protein ubiquitination (Lane 2), but it had no statistical significance when compared with control HepG2 cells. On the other hand, transient overexpression of ISG15 dramatically increased protein ubiquitination (Lane 4). Transfection of cells with the empty plasmid vector had no effect on protein ubiquitination (Lane 3). Equal protein loading was confirmed by probing membranes against an antiactin antibody. Taken together, our results indicated that ectopically overexpressed ISG15 had a different effect compared with IFN-*α*-induced ISG15. Since p53 is one of the most important tumor suppressors involved in the regulation of tumor cell apoptosis, we investigated whether transiently expressed ISG15 and IFN-*α* induced ISG15 had different effects on p53 and p53 downstream protein p21. The expression levels of the p53 and p21 proteins were determined by western blot assay using a specific anti-p53 or anti-p21 antibody. As shown in Figure 2(b), p53 and p21 protein levels in the cells treated with IFN-*α* (Lane 2) were comparable to those of untreated controls (Lane 1) and cells transfected with the empty plasmid vector (Lane 3). However, cells overexpressing ISG15 by transient transfection of the PCDNA3.1-ISG15 vector showed significantly increased p53 and p21 levels (Lane 4). The gray scale protein bands intensity from 4-time experiments was quantified by ImageJ software. Data were analyzed by one-way ANOVA and post hoc LSD test (Figure 2(c)). These results indicated that the upregulation of p53 and p21 expression was specific to the HCC cells with ectopically overexpressed ISG15.

### 3.3. Subcellular Localization of ISG15 in HepG2 Cells

**Figure 3:**
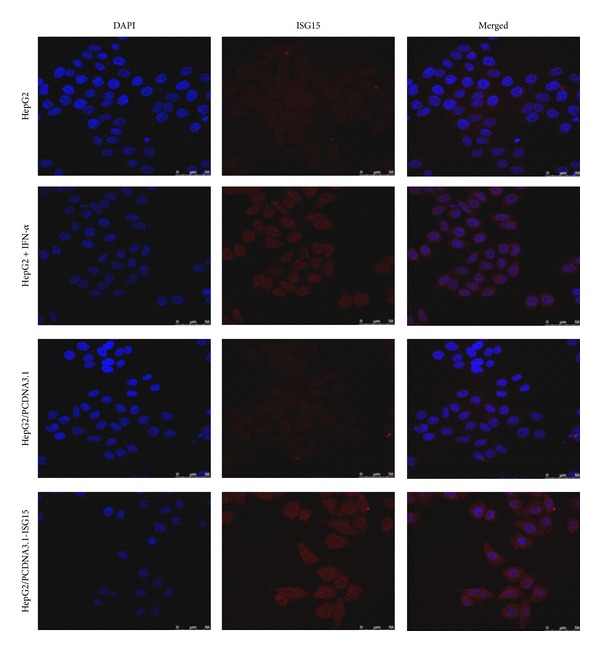
Subcellular localization of ISG15 in HepG2 cells. Free ISG15 and ISG15 modified proteins were detected by confocal-scanning laser microscopy at a magnification of 100x using anti-ISG15 antibody and Cy5-labeled secondary antibody. The Cy5 wavelength was detected by a confocal-scanning laser microscope and is shown in red, while nuclei were stained by DAPI and are shown in blue.

Protein ISGylation plays an important role in the regulation of many cellular pathways, but the exact function and localization of ISG15 and ISG15 modifier proteins are unclear [16]. We used confocal-scanning laser microscopy to determine the subcellular localization of ISG15 in HepG2 cells after 48 h culture. Consistent with the results of western blotting, ISG15 and ISGylated proteins were not detectable in HepG2 cells without IFN-*α* treatment, and the empty plasmid vector transfection had also no effect on ISG15 expression (Figure 3). However, ISG15 was clearly detected when the cells were treated with IFN-*α* or transfected with the PCDNA3.1-ISG15 plasmid. Furthermore, ISG15 localized in both the nucleus and the cytoplasm (Figure 3) suggesting that ISG15 may function in both nucleus and cytoplasm.

### 3.4. Overexpression of ISG15 Inhibits HepG2 Cell Growth

**Figure 4:**
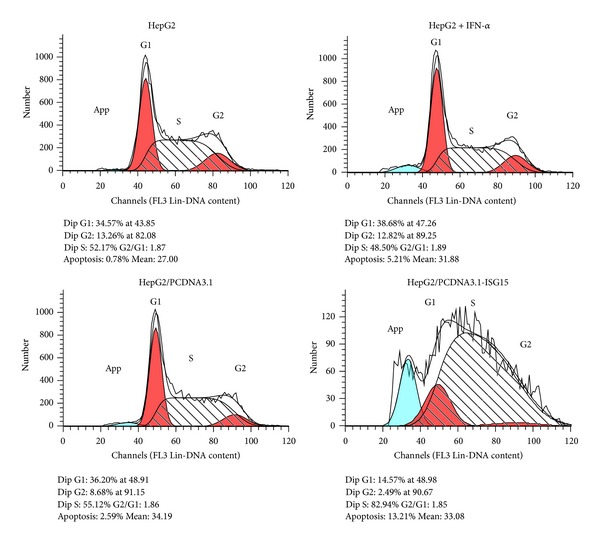
Apoptosis in HepG2 cells under different conditions. HepG2 cells either treated with IFN-*α* (1000 U/mL), transfected with PCDNA3.1, PCDNA3.1-ISG15, or control HepG2 cells were cultured for 48 h. Harvested cells were fixed and treated with 500 *μ*L PBS containing 50 *μ*g/mL PI and 100 *μ*g/mL RNase A at 37°C avoiding light for 30 min. Apoptosis and cell cycle progression were analyzed by flow cytometry (single determination).

Proteasome inhibitors such as MG132 can increase the level of protein ubiquitination and cell apoptosis [17]. Therefore, we investigated whether overexpressed ISG15 had an effect on IFN-*α* resistance and possibly enhanced cancer cell apoptosis by upregulating protein ubiquitination. Flow cytometry was used to determine the distribution of cells in different stages of the cell cycle and detect apoptotic cells. As shown in Figure 4, the percentage of cells in the S phase was 52.17% in control HepG2 cells, 48.5% in IFN-*α*-treated HepG2 cells, and 55.12% in HepG2 cells transfected with the empty plasmid vector. However, up to 82.94% of cells were in the S phase when ISG15 was transiently overexpressed, suggesting that ISG15 overexpression inhibits HepG2 cell growth by inducing S phase arrest. Furthermore, 0.78% and 2.59% of apoptotic cells were detected in control and empty vector transfected cells, respectively, while induction of ISG15 expression by IFN-*α* treatment increased the proportion of apoptotic cells to 5.21%, and overexpression of ISG15 by transient transfection induced apoptosis in 13.21% of HepG2 cells. This single determination indicated that transiently overexpressed ISG15 was more efficient than that induced by IFN-*α* for cell cycle regulation and induction of apoptosis in HepG2 cells, and this explained that the growth of HepG2 cells transfected with ISG15 was slower than the cells treated with IFN-*α* or transfected with control plasmid observed by microscope.

## 4. Discussion

IFN-*α*, as a therapeutic reagent with antitumor properties, has been widely used in combination with other drugs against many malignancies including HCC. However, the clinical outcomes of IFN-*α* therapy have not been satisfactory [18]. More importantly, resistance to IFN-*α* therapy among HCC patients has been reported frequently [19], and the underlying mechanisms of IFN-*α* resistance remain unclear. There are different reasons for IFN-*α* resistance in HCC. For example, mutation or deletion of the IFN alpha receptor 2 (IFNAR2) can lead to the alterations of IFN-*α*-mediated transcription, which ultimately causes the IFN-*α* resistance [20]. Also, there are some reports that indicate the IFN-*α* function independent of IFNAR2. Transcriptional alteration of some genes may cause IFN-*α* resistance too [21]. Moreover, the activation of WNT/*β*-catenin pathway also has role on IFN-*α* resistance in HCC [22]. ISG15 as well as the enzymes involved in ISGylation are upregulated by IFN-*α* presumably through the ISREs in their promoter regions. In the present study, we aimed to gain a better understanding of the anticancer function of IFN-*α* and its targeted gene ISG15. In order to bypass the complex network of IFN-*α*-regulated pathway, we ectopically overexpressed ISG15 in HepG2 cells by transient transfection. This strategy enabled us to compare the similarities and differences between the overexpressed ISG15 and ISGylation in HepG2 cells and that induced by IFN-*α* treatment. Combined with the well-established functions of some molecules, such as ISG15, protease Ubp43 triggered by IFN-*α* could reverse the functions of IFN-*α* by inhibiting the STAT pathway [23]. We prove that IFN-*α*-induced ISG15 did not have an anticancer function, whereas transiently overexpressed ISG15 significantly inhibited HepG2 cell growth and enhanced cell apoptosis. Although these experiments were conducted in HCC cells, the results may be applicable to other IFN-*α*-resistant malignancies and may be helpful to the design of efficient therapeutic strategies for IFN-*α*-resistant carcinoma.

ISG15 has two functional domains, the N-terminal and the C-terminal domains which bear 33% and 32% sequence similarity to ubiquitin, respectively [10]. Both protein ubiquitination and ISGylation processes are the same and can share some common enzymes. The levels of ISGylation and ubiquitination are dynamically regulated by the addition and removal of modifier enzymes. The enzymes such as Usp2, Usp5, Usp13, and Usp14 are responsible for de-ISGylation and de-ubiquitination [24]; however, Ubp43 is only responsible for the removal of ISG15 from ISGylated targets. Because some modifier enzymes can be induced by IFN-*α*, our results showing different effects of IFN-*α*-induced or transiently transfected ISG15 could be dependent on the expression of ISG15 modifier enzymes. IFN-*α* is capable of inducing de-ISGylation enzyme expression and reversing the biological function of ISG15. In the absence of IFN-*α*-induced de-ISGylation enzyme expression, ISG15 increases protein ubiquitination and ISGylation (Figure 2). Therefore, overexpression of ISG15 may affect protein ISGylation directly and protein ubiquitination indirectly. Ubiquitinated proteins play critical roles in various cellular processes such as protein degradation, cell cycle regulation, and DNA replication [25, 26]. Polyubiquitination targets proteins for degradation via the ubiquitin proteasome pathway (UPP), which is a method of eliminating misfolded or damaged proteins in eukaryotes [8]. Blocking the UPP results in the accumulation of ubiquitinated proteins and was shown to inhibit tumorigenesis. Our results indicate that overexpressed ISG15 can increase protein ISGylation and ubiquination, but the IFN-*α* cannot increase the protein ubiquination (Figure 2). These results together with the finding that HCC cell apoptosis was increased only in the cells overexpressing ISG15 (Figure 4) suggest that ISG15 acts by a combination of these effects and its proapoptotic role is mediated by the induction of the accumulation of ubiquitinated proteins.

The p53 is a short-lived protein that is degraded by the UPP [27]. As a key regulator protein, p53 determines cell fate under diverse stress conditions [14]. We examined the possible involvement of p53 in HepG2 cell apoptosis induced by ISG15 overexpression by estimating the levels of the p53 protein in different experimental settings. As shown in Figure 2(c), p53 levels were clearly elevated in HepG2 cells when ISG15 was overexpressed by transient transfection. Although ISG15 levels in response to IFN-*α* induction and transient transfection were comparable, ISG15 upregulated by IFN-*α* treatment failed to modulate p53 levels. The fact that p53 can be modified by ubiquitin [28] together with our findings that overexpression of ISG15 enhanced ubiquitination and apoptosis suggest that overexpression of ISG15 increases cancer cell apoptosis through a p53-dependent pathway. The p21 is the most important downstream protein regulated by the activated p53, which can directly inhibit cell growth, increase apoptosis, and cause cell cycle arrest at S phase [29]. As shown in Figure 2(b), the p21 expression was increased in the cells with ISG15 transient transfection, and it caused HepG2 cell apoptosis and S phase arrest (Figure 4). However, there were multiple factors involved in regulating the cellular levels of p53 and p21; therefore, we cannot ascertain that the changes in p53 or p21 levels in our study were directly caused by the overexpressed ISG15. Mechanistically, ectopic overexpression of ISG15 can bypass the pluripotent effects of IFN-*α* and upregulate p53 and p21 as well as increase HepG2 cell apoptosis efficiently. Although the molecular mechanisms underlying the anticancer effects of ISG15 need to be further explored, our current findings shed light on the roles of ISG15 and provided information essential for its development into an efficient gene therapeutic tool for the treatment of IFN-*α*-resistant HCC.

## 5. Conclusion

The main findings of the present study are as follows: (1) the ectopically expressed ISG15 increases overall protein ubiquitination, upregulates p53 and p21 expression, and ultimately enhances HCC cell apoptosis; (2) ISG15 either induced by IFN-*α* or transiently overexpression localizes in both nucleus and cytoplasm; and (3) overexpression of ISG15 could be developed into an efficient therapeutic strategy for IFN-*α*-resistant HCC and other cancers.
